# Quercetin and doxorubicin co-encapsulated biotin receptor-targeting nanoparticles for minimizing drug resistance in breast cancer

**DOI:** 10.18632/oncotarget.8607

**Published:** 2016-04-06

**Authors:** Li Lv, Chunxia Liu, Chuxiong Chen, Xiaoxia Yu, Guanghui Chen, Yonghui Shi, Fengchao Qin, Jiebin Ou, Kaifeng Qiu, Guocheng Li

**Affiliations:** ^1^ Guangdong Provincial Key Laboratory of Malignant Tumor Epigenetics and Gene Regulation, Sun Yat-Sen Memorial Hospital, Sun Yat-Sen University, Guangzhou 510120, Guangdong, China; ^2^ Department of Pharmacy, Sun Yat-Sen Memorial Hospital, Sun Yat-Sen University, Guangzhou 510120, Guangdong, China; ^3^ Department of Pharmacy, Zengcheng District People's Hospital of Guangzhou, Guangzhou 511300, Guangdong, China

**Keywords:** biotin, doxorubicin, quercetin, co-delivery, drug resistance

## Abstract

The combination of a chemotherapeutic drug with a chemosensitizer has emerged as a promising strategy for cancers showing multidrug resistance (MDR). Herein we describe the simultaneous targeted delivery of two drugs to tumor cells by using biotin-decorated poly(ethylene glycol)-b-poly(ε-caprolactone) nanoparticles encapsulating the chemotherapeutic drug doxorubicin and the chemosensitizer quercetin (BNDQ). Next, the potential ability of BNDQ to reverse MDR *in vitro* and *in vivo* was investigated. Studies demonstrated that BNDQ was more effectively taken up with less efflux by doxorubicin-resistant MCF-7 breast cancer cells (MCF-7/ADR cells) than by the cells treated with the free drugs, single-drug–loaded nanoparticles, or non-biotin–decorated nanoparticles. BNDQ exhibited clear inhibition of both the activity and expression of P-glycoprotein in MCF-7/ADR cells. More importantly, it caused a significant reduction in doxorubicin resistance in MCF-7/ADR breast cancer cells both *in vitro* and *in vivo*, among all the groups. Overall, this study suggests that BNDQ has a potential role in the treatment of drug-resistant breast cancer.

## INTRODUCTION

Chemotherapy is one of the major treatments in clinical cancer therapy, but the emergence of multidrug resistance (MDR) is the biggest obstacle to successful chemotherapy [1–7]. Overexpression of ATP-binding cassette (ABC) transporters such as P-glycoprotein (P-gp) is one of the main and best understood causes of MDR [8, 9]. ABC transporters act as drug efflux pumps, which can dramatically extrude many antitumor drugs from the target cancer cells, and then reduce the intracellular drug concentration, so limiting the anticancer effect of the drug in tumors. To achieve therapeutic concentrations in cancer cells, progressively higher dosages of drugs are needed [3]. However, such higher dosages of drugs may cause cytotoxicity to normal cells, such as the severe toxicity shown by doxorubicin (DOX) to the heart. To overcome these problems, one of the best strategies is the development of active tumor-targeted drug delivery systems for the co-delivery of anticancer drugs and ABC transporter inhibitors. These drug delivery systems could enhance the delivery of anticancer drug to the tumor and inhibit the function of drug efflux transporters.

A variety of P-gp inhibitors such as verapamil and cyclosporine have been reported to overcome MDR, however these agents have many side effects [10, 11]. In order to search for less toxic MDR-reversing agents, a number of natural products with less toxicity were investigated as P-gp inhibitors. Quercetin (QUT) is a flavonoid present in many edible fruits and vegetables such as tea, grape, tomato, and apple [12, 13]. It is now extensively used as a dietary supplement and marketed in the United States [12, 14]. It has been reported that QUT can interact directly with several ABC transporter proteins to inhibit drug efflux. Furthermore, QUT can inhibit the expression of P-gp and MDR1 gene products. Therefore, QUT can function as a chemosensitizer to enhance the cytotoxic effects of chemotherapeutic drugs on many cells showing MDR such as MCF-7/ADR cells, MCF/MR cells, and K562/BCRP cells [8, 10, 12, 14–19]. Despite these promising properties of QUT, there is a long way to go before QUT comes into application in the clinical setting. A severe limitation to the introduction of QUT in clinical trials is its extremely low aqueous solubility [20–22].

The use of polymeric drug delivery systems such as polymeric nanoparticles (NPs) is considered to be one of the most promising strategies for overcoming the poor water solubility of drugs [23–27]. There are several advantages of NPs compared to traditional free chemotherapeutic drugs. Firstly, NPs allow higher tumor accumulation of encapsulated drugs by the enhanced permeability and retention (EPR) effect [28]. Secondly, NPs may further increase the tumor-targeted delivery by modification of the surface of NPs with specific tumor cell targeting ligands, such as folic acid, biotin, and antibodies [29–32]. Additionally, NPs can deliver multiple types of agents with different anticancer mechanisms such as a chemotherapeutic drug with a chemosensitizer, to exert synergistic anticancer effects [33–36].

Herein, biotin-decorated poly(ethylene glycol)-b-poly(ε-caprolactone) nanoparticles encapsulating the chemotherapeutic drug DOX and the chemosensitizer QUT (BNDQ) were prepared. Then the potential effect of BNDQ in minimizing drug resistance of MCF-7/ADR tumor was investigated and the underlying mechanisms were also explored.

## RESULTS AND DISCUSSION

### Characterization of the drug-loaded nanoparticles

**Figure 1:**
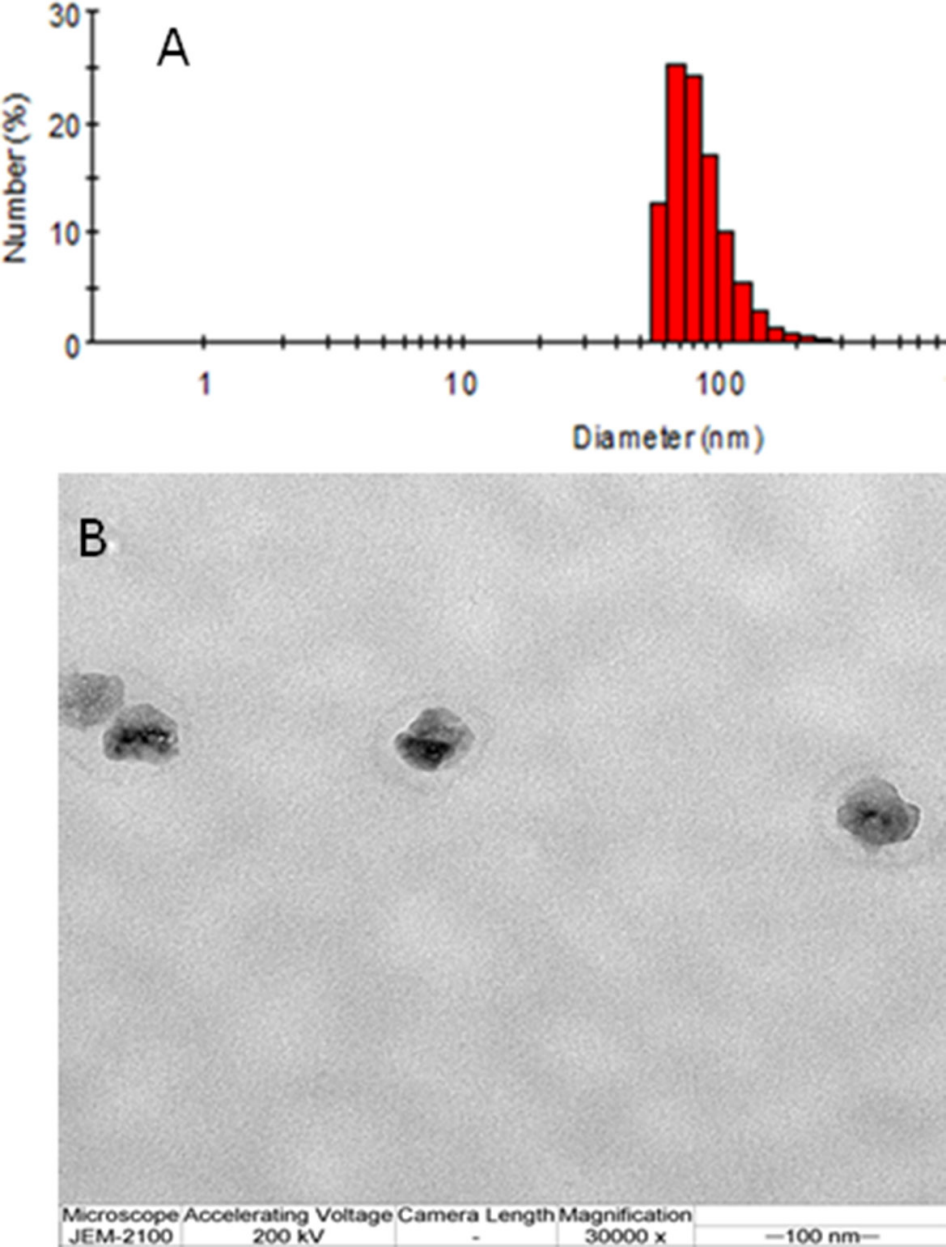
The hydrodynamic diameters by DLS (A) and the TEM image (B) of the BNDQ

The nanoparticles were prepared using a thin-film hydration method. As shown in Table 1 and Figure 1, the particle sizes were 90.2 ± 2.4, 95.6 ± 1.4, 100.2 ± 2.8, and 105.8 ± 1.4 nm for methoxy poly(ethylene glycol)-b-poly(ε-caprolactone) nanoparticles encapsulating doxorubicin (MND), methoxy poly(ethylene glycol)-b-poly(ε-caprolactone) nanoparticles encapsulating QUT (MNQ), methoxy poly(ethylene glycol)-b-poly(ε-caprolactone) nanoparticles encapsulating doxorubicin and QUT (MNDQ), and BNDQ, respectively, as assessed by dynamic light scattering (DLS). The sizes of the nanoparticles are of critical importance since particle size not only affects the endocytosis behavior of tumor cells, but also influences the accumulation effect of nanoparticles in tumor tissue *via* the EPR effect. They should be normally within the range of 8-200 nm [37–39]. In this study, the hydrodynamic sizes of all prepared nanoparticles were within the range of 8–200 nm, suggesting they could be beneficial in targeted tumor delivery. The polydispersity indexes of the prepared nanoparticles were < 0.20. Transmission electron microscopy (TEM) was used to directly visualize the morphology and size of BNDQ (Figure 1B). Approximately spherical morphology and moderate uniform size distribution were observed for BNDQ. The zeta-potential of the prepared nanoparticles were from −7.68 to −9.56 mV, indicating that the surface charge of the prepared nanoparticles was negative. A slightly negative charge of the nanoparticle could reduce reticuloendothelial system (RES)-mediated clearance, consequently enhancing the blood circulation time. The drug loading contents of all the prepared nanoparticles were all above 3.5%, and the encapsulation efficiencies were all higher than 80%.

**Table 1:** Particle size, size distribution, surface zeta potential, drug loading and encapsulation of the prepared nanoparticles

Formulation	Size (nm)	PDI^a^	Zeta potential (mV)	Drug loading content (%)	Encapsulation efficiency (%)
MND	90.2 ± 2.4	0.113 ± 0.020	−8.02 ± 0.18	4.0	90
MNQ	95.6 ± 1.4	0.168 ± 0.024	−7.68 ± 2.24	7.9	92
MNDQ	100.2 ± 2.8	0.175 ± 0.009	−9.12 ± 1.60	3.8^b^, (7.8^c^)	88 ^b^, (90^c^)
BNDQ	105.8 ± 1.4	0.168 ± 0.023	−9.56 ± 1.80	3.6^b^, (7.9^c^)	86^b^, (91^c^)

PDI^a^: polydispersity index; ^b^for DOX; ^c^ for QUT.

MND: DOX-loaded MPEG-PCL nanoparticle; MNQ: QUT-loaded MPEG-PCL nanoparticle; MNDQ: DOX and QUT-loaded MPEG-PCL nanoparticle; BNDQ: DOX and QUT-loaded Biotin-PEG-PCL nanoparticle.

### *In vitro* drug released from the drug-loaded nanoparticles

**Figure 2:**
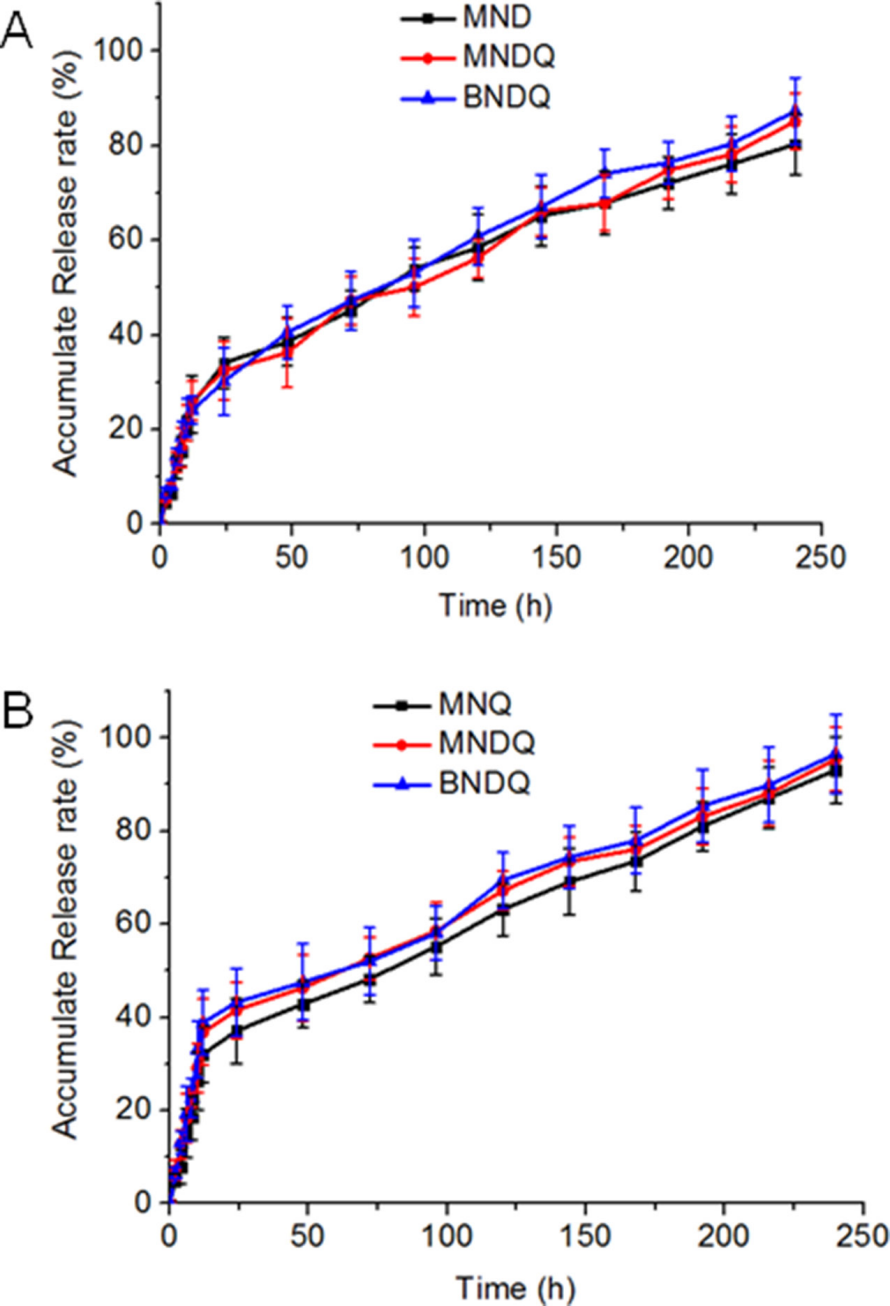
*In vitro* release profiles of the drug-loaded nanoparticles in PBS (0.01 M, pH = 7.4 containing 0.5% of Tween 80) (**A**) DOX release from MND, MNDQ and BNDQ; (**B**) QUT release from MND, MNDQ and BNDQ.

In this study, pH 7.4 phosphate buffer solution was selected to simulate the blood environment. The release profiles of DOX and QUT from the drug-loaded nanoparticles are shown in Figure 2. The release profiles of DOX from both the single drug-loaded nanoparticles (MND) and dual drug-loaded nanoparticles (MNDQ or BNDQ) exhibited biphasic patterns of drug release. After the initial burst release over about 12 h, the release rate of DOX slowed down to show sustained release patterns. During the first 12 h, the percentages of DOX released from the MMD, MNDQ, and BNDQ were 25.34%, 26.12%, and 24.12%, respectively. This may be attributed to the diffusion of DOX that was adsorbed on the surface of the nanoparticle. After 240 h, approximately 80.34%, 85.12%, and 87.34% of the total DOX was found to be released from MMD, MNDQ, and BNDQ, respectively. As the drugs slowly diffused from the nanoparticle core, the DOX-loaded nanoparticles showed sustained drug release profiles. The release of QUT from MNQ, MNDQ, and BNDQ followed a similar release pattern to that of DOX.

### *In vitro* cytotoxicity studies in drug sensitive and drug resistant cells

**Figure 3:**
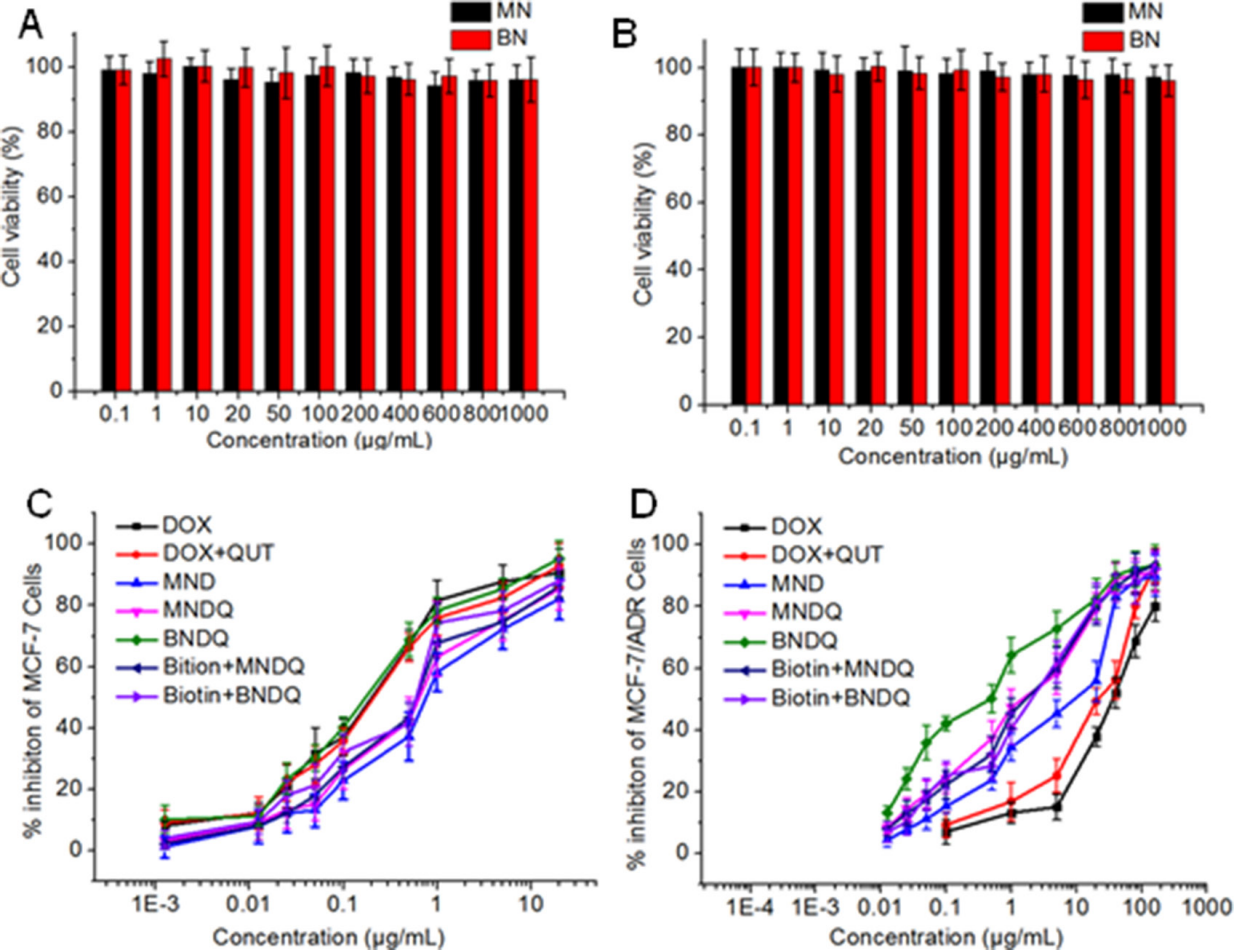
*In vitro* cytotoxicities of empty nanoparticles and different DOX formulations against MCF-7 and MCF-7/ADR cells for 48 h (**A**) MCF-7 cells were treated with empty nanoparticles; (**B**) MCF-7/ADR cells were treated with empty nanoparticles; (**C**) MCF-7/ADR cells were treated with DOX, (DOX+QUT), MND, MNDQ, BNDQ, (free Biotin + MNDQ) or (free Biotin + BNDQ); (**D**) MCF-7/ADR cells were treated with DOX, (DOX + QUT), MND, MNDQ, BNDQ, (free Biotin + MNDQ) or (free Biotin + BNDQ).

The *in vitro* cytotoxicities of all the drug formulations were investigated in drug-sensitive MCF-7 cells and P-gp-overexpressing MCF-7/ADR cells by using the 3-(4,5-dimethylthiazol-2-yl)-2,5-diphenyltetrazolium bromide (MTT) assay. First, the cytotoxicities of the empty nanoparticles were investigated to study the safety of the drug carriers. As shown in Figure 3A and 3B, after being treated with the empty methoxy poly(ethylene glycol)-b-poly(ε-caprolactone) (MPEG-PCL) or biotin-decorated poly(ethylene glycol)-b-poly(ε-caprolactone) (biotin-PEG-PCL) nanoparticles over the concentration range of 0.1 to 1000 μg/mL, the cell viabilities of both the MCF-7 and MCF-7/ADR cells were all above 90%. These results suggest that the carriers used in this study are non-toxic at such concentrations. Next, the cytotoxic activities of free drug and drug-loaded nanoparticles were investigated to study the abilities of drug formulations to minimize drug resistance. As shown in Figure 3C and 3D, BNDQ exhibited remarkably higher cytotoxicity than that exhibited by the combination of the free drugs (DOX + QUT) or non-biotin decorated nanoparticles (MNDQ) at all drug concentrations in both MCF-7 and MCF-7/ADR cells. The IC_50_ value (the concentration that inhibited cell growth by 50%) of BNDQ in MCF-7/ADR cells was 0.26 μg/mL, which was 136, 94-, 31- and 5-fold less than that in the cells incubated with DOX, (DOX+QUT), MND, and MNDQ respectively. Further, in MCF-7 cells, the IC_50_ value of BNDQ was found to be 0.05, 0.12, 3.64, and 2.52 times lower than that of DOX, (DOX+QUT), MND and MNDQ, respectively (Table 2). We hypothesized that the enhanced cytotoxicity of BNDQ over MNDQ is attributed to higher cellular uptake of BNDQ. To test this hypothesis, we studied the free biotin inhibition effect on the *in vitro* cytotoxicities of BNDQ and MNDQ. It was found that the IC_50_ of BNDQ was significantly enhanced by the presence of free biotin compared to that in the absence of biotin in both MCF-7 and MCF-7/ADR cells, while that for the MNDQ was statistically unaffected (Table 2). This clearly indicated that the enhanced cytotoxicity associated with BNDQ might be due to biotin receptor mediated endocytosis. This hypothesis was further demonstrated in the following drug cellular uptake study.

**Table 2:** The IC_50_ of DOX in MCF-7 and MCF-7/ADR cells, index of drug resistance (IDR) and index of reversal of drug resistance (IRDR) of DOX in MCF-7/ADR cells treated with different formulations

Formulation	MCF-7		MCF-7/ADR	
	IC_50_(μg/mL)	IC_50_(μg/mL)	^a^IDR	^b^IRDR
DOX	0.18	35.63	197.94	—
DOX + QUT	0.19	24.83	137.94	1.43
MND	0.79	8.28	46.00	4.30
MNDQ	0.60	1.62	9.00	21.99
BNDQ	0.17	0.26	1.44	137.45
Biotin + MNDQ	0.53	1.74	9.67	20.47
Biotin + BNDQ	0.58	1.97	10.94	18.09

MND: DOX-loaded MPEG-PCL nanoparticle; MNQ: QUT-loaded MPEG-PCL nanoparticle; MNDQ: DOX and QUT-loaded MPEG-PCL nanoparticle; BNDQ: DOX and QUT-loaded Biotin-PEG-PCL nanoparticle.

To further investigate the differences in the MDR-reversal abilities of drug formulations on MCF-7/ADR cells, the concepts of index of drug resistance (IDR) and index of reversal of drug resistance (IRDR) were introduced. IDR and IRDR were calculated using the following equations:

$$\begin{array}{l} \text{IDR}=\frac{IC_{50}\text{ of other formulation againts MCF }-7/\text{ADR cells}}{IC_{50}\text{ of free DOX against MCF }-7/\text{cells}}\\ \text{IRDR}=\frac{IC_{50}\text{ of free DOX againts MCF }-7/\text{cells}}{IC_{50}\text{ of other formulation againts MCF }-7/\text{ADR cells}} \end{array}$$

The IDR decreased from 197.94 for DOX to, 137.94 for (DOX + QUT), 46.00 for MND, 9.00 for MNDQ, and 1.44 for BNDQ. The IRDR was 1.43, 4.30, 21.99 and 137.45 for (DOX + QUT), MND, MNDQ and BNDQ, respectively, (Table 2). It is worth noting that BNDQ exhibited a stronger ability in reversing drug resistance compared to other formulations, as confirmed by the higher IRDR and the lower IDR.

### *In vitro* cellular uptake studies in drug sensitive and drug resistant cells

**Figure 4:**
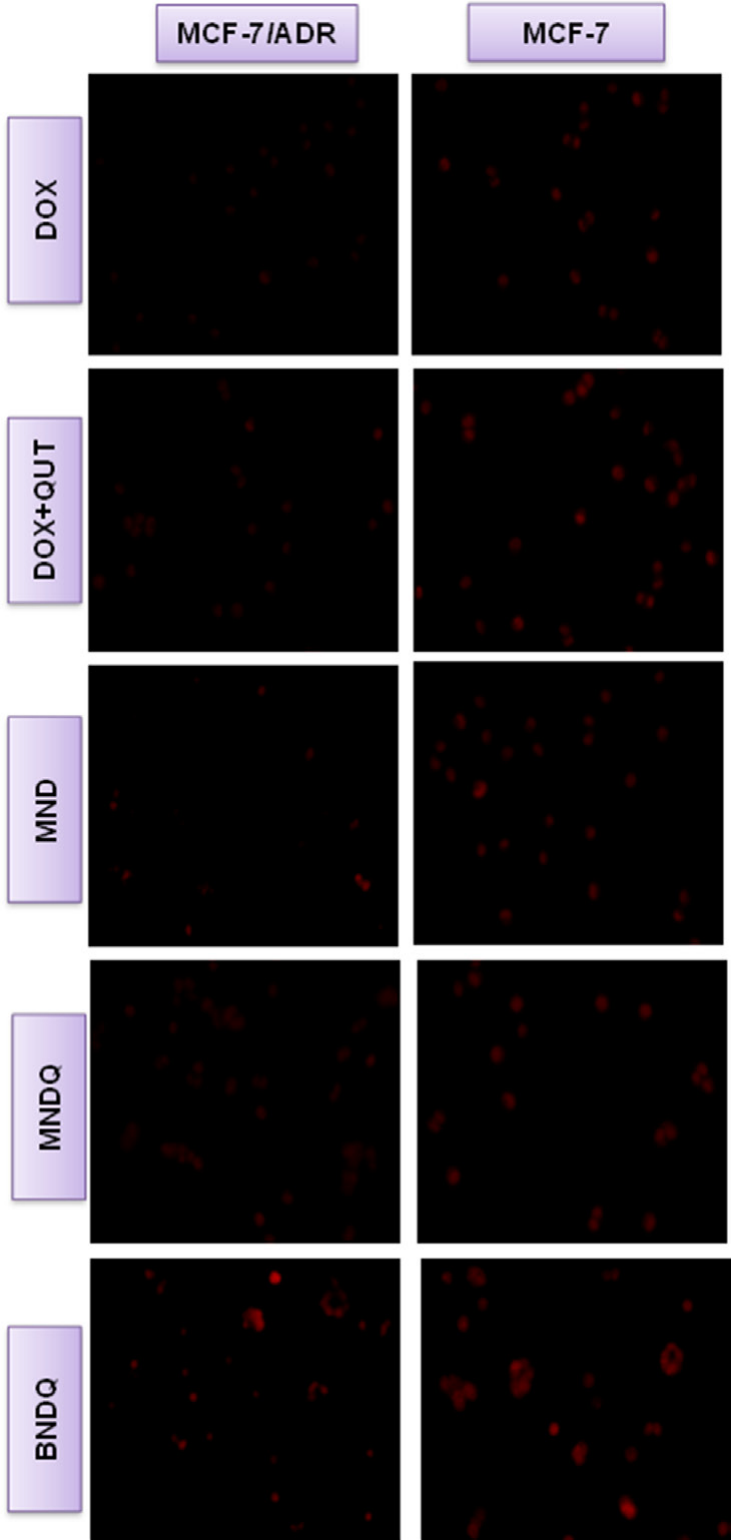
Fluorescent microscopy observations of MCF-7/ADR and MCF-7 cells after incubation with free DOX, DOX + QUT, MND, MNDQ and BNDQ for 4 h

**Figure 5:**
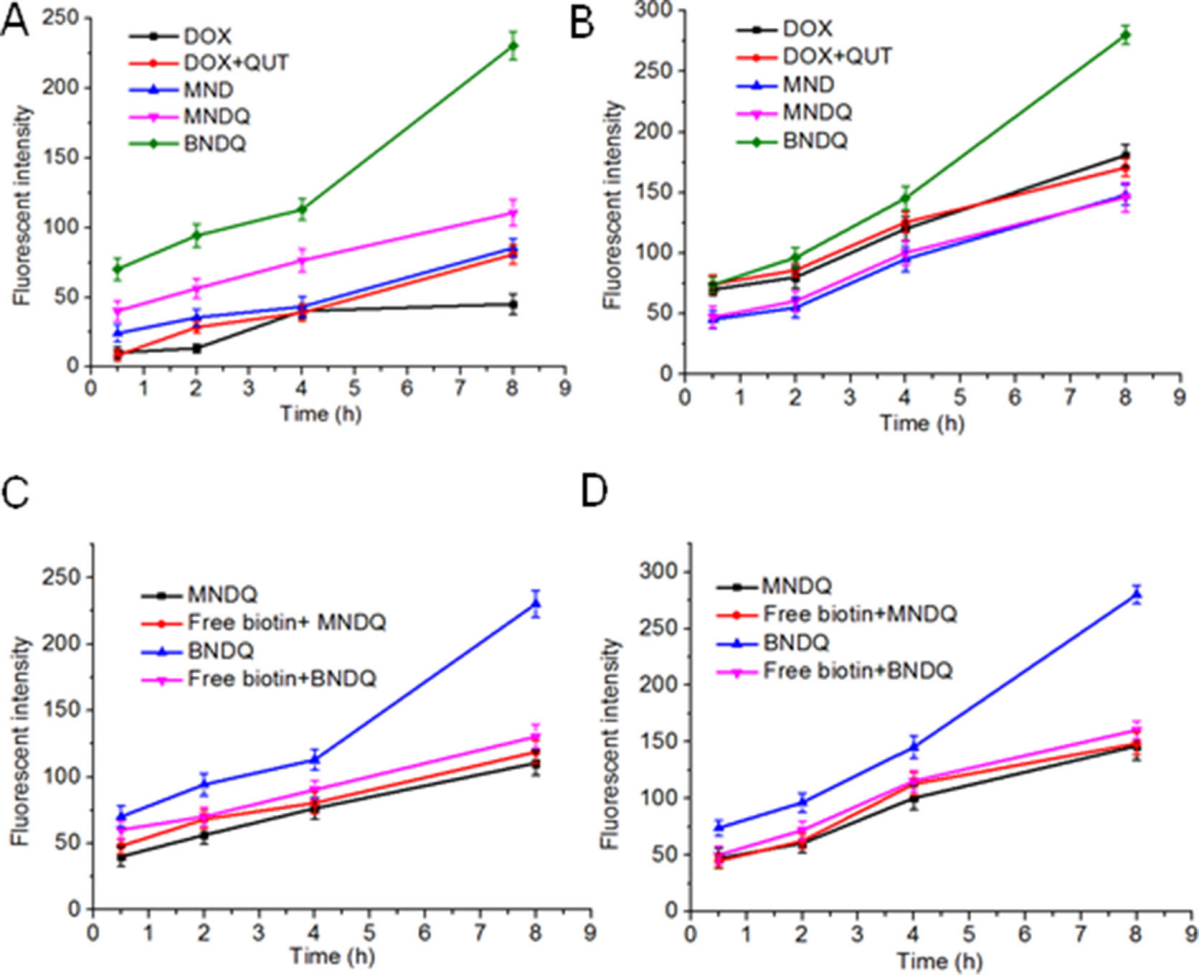
Cellular uptake behaviors of different formulations of DOX in MCF-7 and MCF-7/ADR cells MCF-7/ADR cells (**A**) and MCF-7 cells (**B**) were incubated with different formulations of DOX (DOX, DOX+CUR, MND, MNDQ, BNDQ) for 0.5, 2, 4 and 8 h, respectively. At the end of each incubation time, the fluorescent intensities of cells were measured by flow cytometer. For the competitive experiment in presence of free biotin, MCF-7/ADR cells (**C**) and MCF-7 cells (**D**) were treated with 2 mM of free biotin for 1.5 h and then incubated with new media containing MNDQ or BNDQ for another 0.5, 2, 4 and 8 h.

Firstly, the cellular uptake behaviors of different formulations of DOX were examined in MCF-7 cells and MCF-7/ADR cells with a fluorescence microscope. Figure 4 shows representative fluorescence images of cells after incubation for 4 h with different DOX formulations. As evidenced in the figure, the enhanced uptake of BNDQ was observed in both MCF-7 and MCF-7/ADR cells, compared to MNDQ, MND, (DOX+QUT) and free DOX. Next, the extent of cellular uptake of the drug from the different DOX formulations was further quantitatively determined by flow cytometry. The relative extents of cellular uptake of drug in DOX, (DOX + QUT), MND, MNDQ, BNDQ, free biotin + MNDQ and free biotin + BNDQ are shown in Figure 5 in terms of mean fluorescence intensity (MFI). As shown in Figure 5A and 5B, MFI of both MCF-7 and MCF-7/ADR cells increased with incubation time enhanced from 0.5 h to 8 h, indicating that the cellular uptake of all the DOX formulations in these cells increased in a time-depend manner. The MFIs of MCF-7/ADR cells after 8 h incubation with free DOX, (DOX + QUT), MND, MNDQ and BNDQ were 45.17 ± 7.29, 80.78 ± 7.12, 85.31 ± 6.98, 110.56 ± 9.45 and 230.45 ± 10.21 respectively. The MFI of MCF-7/ADR cells in MNDQ increased significantly, showing 2.4-, 1.4- and 1.3-fold increases in the DOX, (DOX + QUT) and MND formulations, respectively. This may be due to the combination effect of nanoparticles with a chemosensitizer (QUT) in overcoming drug resistance. On the contrary, due to the low expression of P-gp, there were no significant differences in the cellular uptake of drug in MCF-7 cells among the DOX, (DOX + QUT), MND and MMDQ formulations. The cellular uptake of BNDQ was further increased in both MCF-7/ADR and MCF-7 cells. In addition, the MFIs of MCF-7/ADR and MCF-7 cells after incubation with BNDQ were 2.1 and 1.9 times that of MNDQ, respectively. These results clearly indicate that the superior drug uptake seen in BNDQ in comparison to MNDQ is attributed to biotin receptor mediated endocytosis.

To verify that the uptake of BNDQ is mediated *via* the biotin receptor, a competition experiment was performed. Free biotin (2 mM) was used as a competitive inhibitor in the experiment. As shown in Figure 5C and 5D, 2 mM free biotin significantly reduced the cellular uptake of BNDQ (biotin decorated NPs) in both MCF-7 and MCF-7/ADR cells compared with those without free biotin competition. However, it did not reduce the cellular uptake of MNDQ (non-biotin decorated NPs) in both MCF-7 and MCF-7/ADR cells compared with those without free biotin competition. These results showed that the cellular uptake of BNDQ could be inhibited by the presence of free biotin, and further demonstrated that BNDQ may be endocytosed *via* the biotin receptor.

### *In vitro* drug efflux study

**Figure 6:**
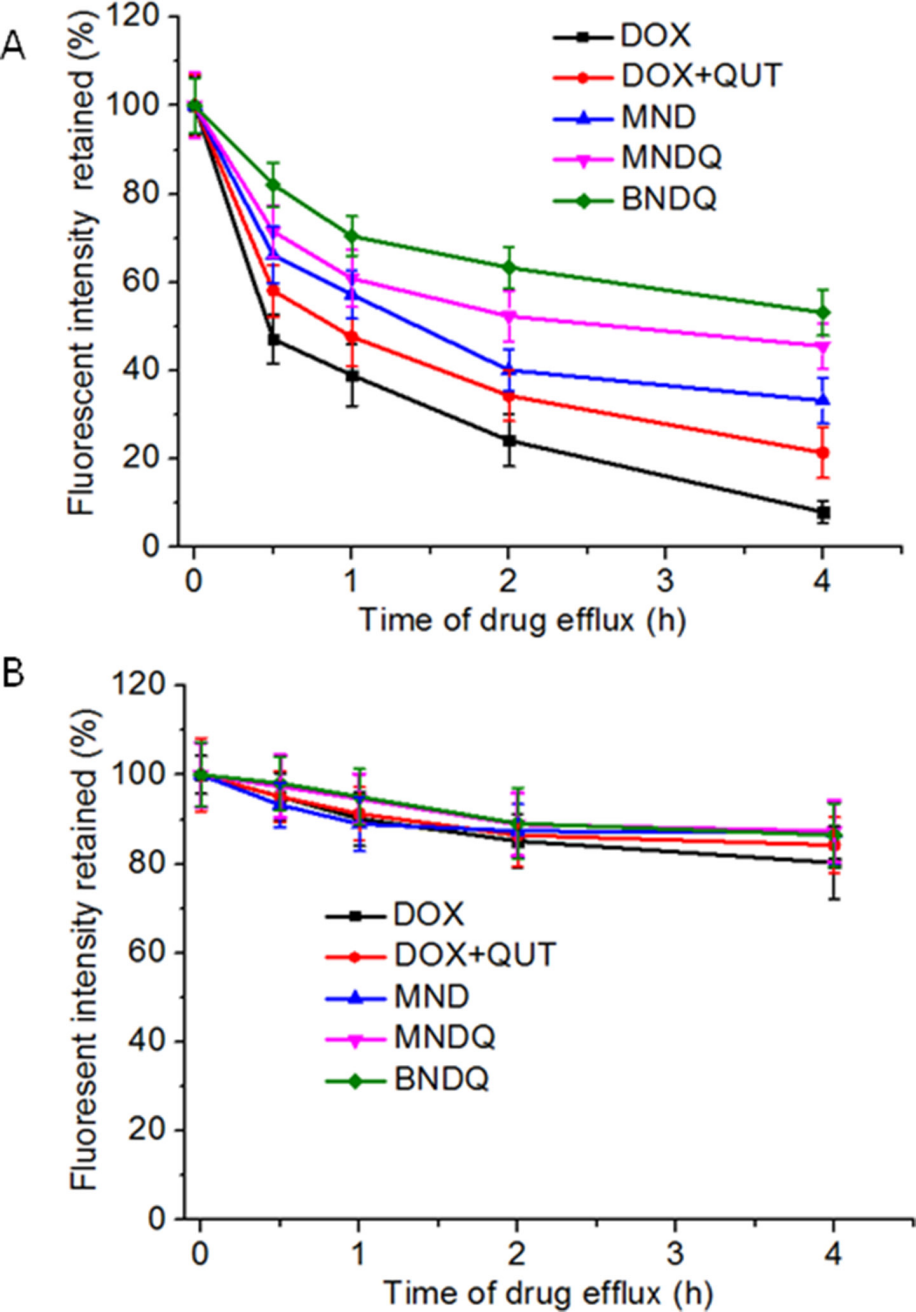
DOX retained in the MCF-7/ADR cells (**A**) and MCF-7 cells (**B**) after treated with free DOX, (DOX + QUT), MND, MNDQ and BNDQ. The cells were incubated with media containing different DOX formulations for 4 h at 37°C. After washing the cells with PBS, cells were incubated with drug-free media for another 0.5, 1, 2 and 4 h. The amounts of DOX retained in the cells were determined by flow cytometer.

MCF-7 or MCF-7/ADR cells were first cultured with DOX, (DOX + QUT), MND, MNDQ or BNDQ for 4 h at 37°C. After washing three times with PBS to remove extracellular drug, the rinsed cells were incubated with fresh drug-free media for another 0.5, 1, 2, or 4 h. The amount of DOX retained in cells was determined by flow cytometer to measure the drug efflux (Figure 6). After incubation of 4 h, 92%, 79%, 67%, 55% and 47% of DOX were effluxed in the DOX, (DOX + QUT), MND, MNDQ and BNDQ treated MCF-7/ADR cells, respectively. The highest efflux rate in the DOX group may be attributed to the pumping out of DOX by P-gp. In (DOX + QUT) group, the efflux rate was significantly slower, compared to the DOX group, because of the P-gp inhibition effect of QUT. The efflux rates were further decreased when DOX and QUT were co-encapsulated in nanoparticles (MNDQ or BNDQ group), due to the combination effect of nanoparticle and QUT in minimizing drug efflux. In MCF-7 cells, due to the lower expression of P-gp, the cellular concentration of DOX in the initial stage of the test was remarkably higher than that in MCF-7/ADR cells; no significant drug efflux was seen during the study period any of the DOX formulations-treated groups.

### The nanoparticle effects on P-gp activity and expression

**Figure 7:**
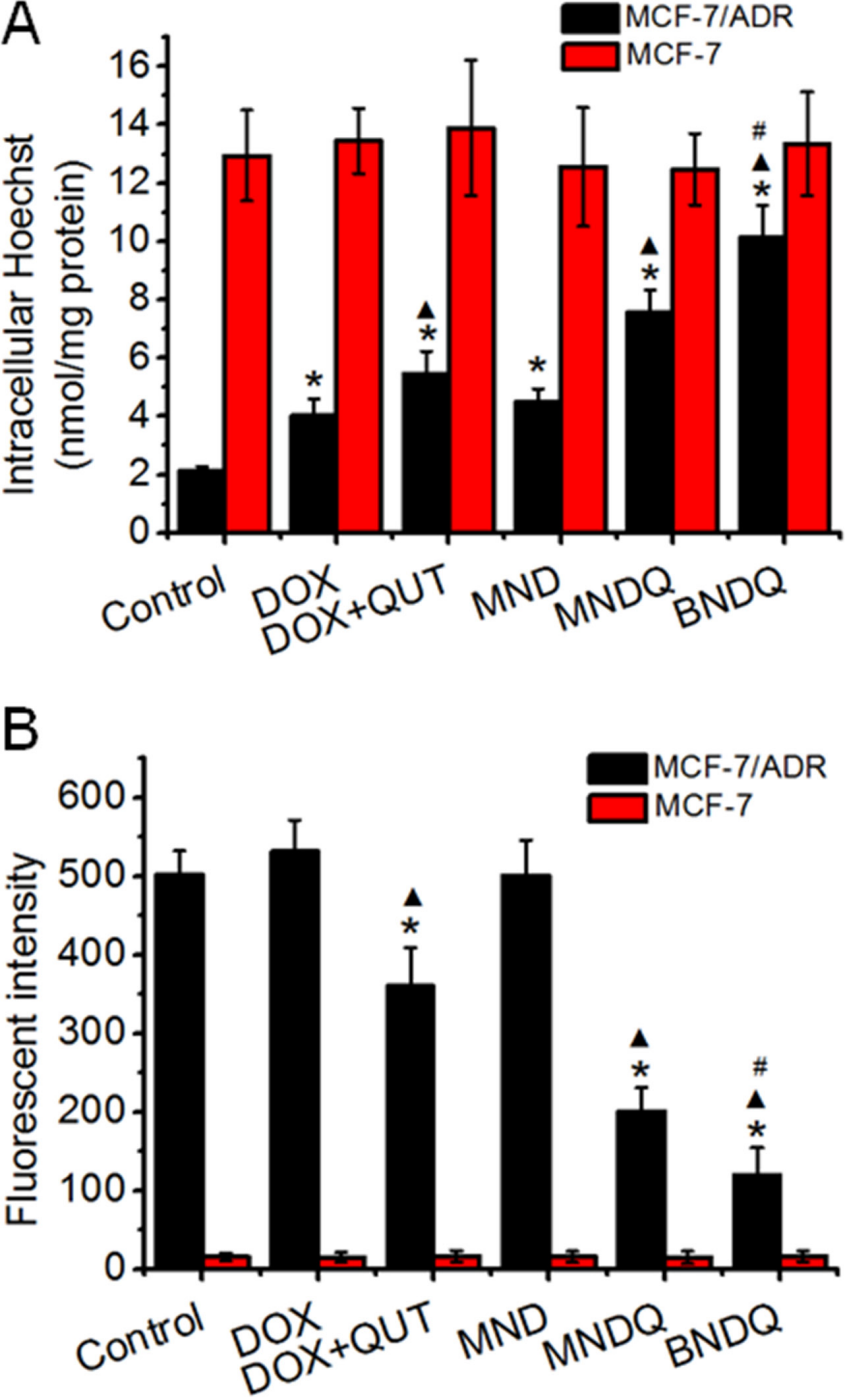
The P-gp activity (**A**) and expression (**B**) of MCF-7/ADR and MCF-7 cells after treatment with free DOX, (DOX + QUT), MND, MNDQ and BNDQ. In the experiment of P-gp activity, the cells were incubated for 4 h in the drug-free medium (control), free DOX, (DOX + QUT), MND, MNDQ or BNDQ, and Hoechst 33342 (4 μM) were added to the culture media and incubated for another 0.5 h. Then the cells were washed, lysed and analyzed fluorimetrically for the intracellular content of the dye. The dye concentration was standardized by the protein concentration which was measured with BCA assay. In the experiment of P-gp expression, the cells were cultured with the drug-containing media or drug-free media (control group) for 48 h, and then the cells were trypsinized, collected, and resuspended. PE-conjugated mouse anti-human monoclonal antibody against P-gp was use to label above cells, and the fluorescent intensities of the cells were determined. Measurements were performed in triplicate and data are presented as mean ± SD (*n* = 3). (*p < 0.01, compared to control group; ^▴^*p < 0.01*, compared to DOX group; ^#^*p < 0.01*, compared to MNDQ group).

The P-gp function and the expression of P-gp in both MCF-7/ADR and MCF-7 cells were analyzed, after treatment with different DOX formulations, to study whether co-encapsulation of DOX and QUT into the same nanoparticle could enhance QUT's ability to inhibit P-gp activity and expression. Hoechst 33342, a specific substrate of P-gp, was used to analyze P-gp activity in the cancer cells after treatment with DOX, (DOX + QUT), MND, MNDQ or BNDQ. As shown in Figure 7A, all of the DOX formulations could significantly enhance the accumulation of Hoechst 33342 in MCF-7/ADR cells. The intracellular concentrations of Hoechst 33342 were increased 1.9, 2.6-, 2.1-, 3.6- and 4.8-fold compared to control after treatment with DOX, (DOX + QUT), MND, MNDQ and BNDQ, respectively. In the (DOX + QUT)-treated group, the accumulation of Hoechst 33342 was 2.6-fold higher compared to the control group and 1.4-fold higher than the DOX-treated group. This result demonstrated that QUT can inhibit P-gp activity. When DOX and QUT were coencapsulated into the same nanoparticle forming MNDQ or BNDQ, the accumulation of Hoechst 33342 increased 3.6-fold compared to control in the MNDQ-treated group and 4.8-fold to control in the BNDQ-treated group. These results may be, at least partially, attributable to the significant increase in cellular uptake of (QUT + QUT) in MCF-7/ADR cells after DOX and QUT were co-encapsulated into the same nanoparticle. However, none of the DOX formulations altered the Hoechst accumulations in the drug sensitive MCF-7 cells significantly.

The levels of P-gp expression were detected by flow cytometer using a fluorescence-labeled anti-MDR1 antibody after the cells were treated with different DOX formulations for 48 h. As shown in Figure 7B, MFIs of MCF-7/ADR cells were were 106% ± 8%, 72% ± 10%, 99% ± 8%, 40% ± 7% and 24% ± 7% of the control group, after treatment with DOX, (DOX + QUT), MND, MNDQ and BNDQ, respectively.

From the results of DOX- and (DOX+QUT)-treated groups, we conclude that free QUT has the ability to down regulate P-gp expression in MCF-7/ADR cells (72% ± 10% of (DOX + QUT)-treated group *vs* 106% ± 8 of DOX-treated group). When the DOX and QUT were co-encapsulated into the same nanoparticle (MNDQ), the inhibition rate of P-gp expression was further increased (40% ± 7% of MNDQ-treated group *vs* 72% ± 10% of (DOX + QUT)-treated group). This may be attributed to the combination effects of QUT and nanoparticles. In the BNDQ-treated group, MCF-7/ADR cells showed a significant decrease in the expression of P-gp, when compared to the MNDQ-treated group (40% ± 7% of MNDQ-treated group *vs* 72% ± 10% of (DOX + QUT)-treated group). After treatment with the blank media, DOX, (DOX + QUT), MND, MNDQ and BNDQ, the fluorescence intensities of MCF-7 cells were 14.89 ± 6.54, 16.34 ± 7.54, 15.56 ± 7.08, 14.97 ± 8.09 and 16.45 ± 7.03, respectively. Owing to the low expression of P-gp in MCF-7 cells, there were no significant differences among all the treatment groups.

### *In vivo* tumor drug accumulation efficacy

**Figure 8:**
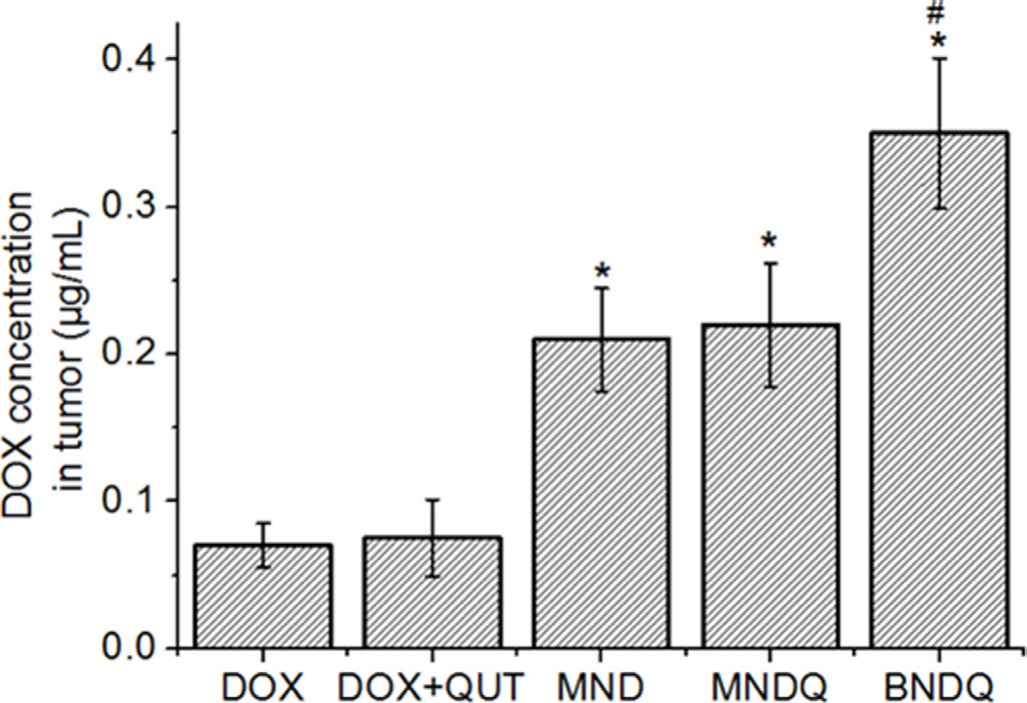
The DOX accumulations in MCF-7/ADR tumor tissues (*n* = 4) after 12 h administration of free DOX, (DOX + QUT), MND, MNDQ or BNDQ, at the DOX dosage of 5 mg/kg (*p < 0.01, compared to DOX group, #*p < 0.01*, compared to MNDQ group).

To evaluate the effect of the nanoparticle system on the *in vivo* tumor accumulation of DOX, the DOX concentrations in tumor tissue were measured after MCF-7/ADR tumor-bearing nude mice were injected with free DOX, (DOX+QUT), MND, MNDQ, or BNDQ. As shown in Figure 8, after 12 h of administration, the DOX concentrations in tumors were 0.07, 0.08, 0.21, 0.22 and 0.35 μg/mL for the injections of free DOX, (DOX + QUT), MND, MNDQ, and BNDQ, respectively. The MND-treated group exhibited a 3.0-fold higher accumulation of DOX in the tumor when compared to the free DOX-treated group. Similarly, when compared to the (DOX + QUT)-treated group, the MNDQ-treated group showed a 2.8-fold higher accumulation in the tumor. These results demonstrated that MND and MNDQ were capable of delivering substantially higher quantity of drugs into tumors. When compared to the MNDQ-treated group (the carrier without biotin decoration), the BNDQ-treated group (the carrier decorated with biotin) showed a 1.6-fold higher accumulation of DOX in tumor, clearly indicating that biotin-decoration of the carrier could further enhance the drug accumulation in tumors. The *in vivo* results were consistent with the results of the *in vitro* cellular uptake study. The results showing the BNDQ-treated group with the highest cellular uptake *in vitro* and the highest tumor accumulation *in vivo* may be attributed to the biotin receptor-targeting ability. There was no significant difference in the DOX concentration in tumor tissue, when comparing the free DOX-treated group with the (DOX + QUT)-treated group. A similar phenomenon was observed comparing the MND-treated and MNDQ-treated groups, demonstrating that QUT had no effect on the accumulation of DOX in tumor tissue.

### *In vivo* antitumor efficacy

**Figure 9:**
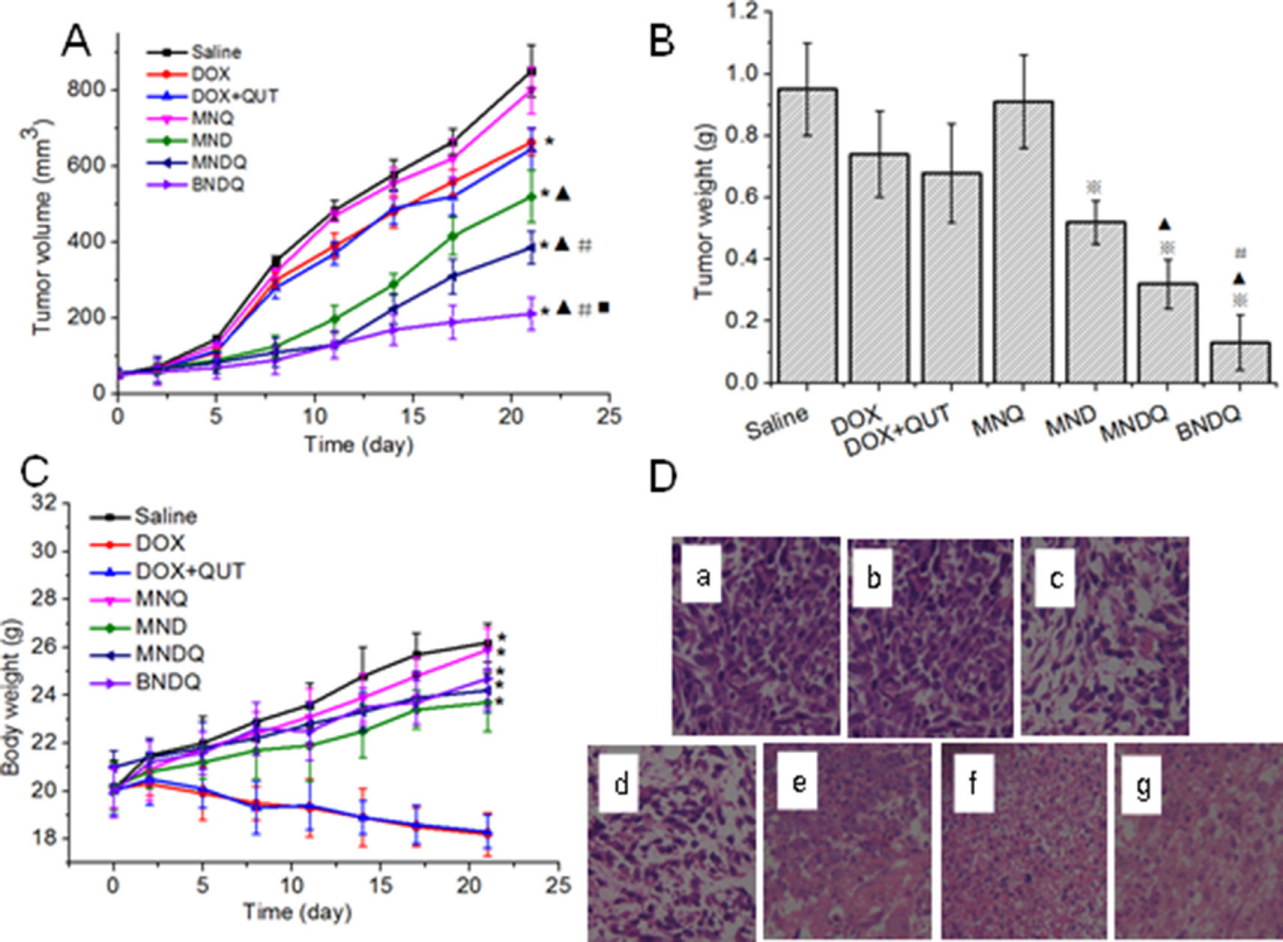
*In vivo* antitumor effect of DOX, (DOX + QUT), MNQ, MND, MNDQ and BNDQ on nude mice bearing MCF-7/ADR tumors (*n* = 6) (**A**) Tumor volume *vs.* time (*p < 0.01, compared to control group; ^▴^*p < 0.01*, compared to DOX group; ^#^*p < 0.01*, compared to MND group; ^◾^*p < 0.01*, compared to MNDQ group); (**B**) Tumor weights at the end of the experiment (^❄^*p < 0.01*, compared to DOX group;^▴^*p < 0.01*, compared to MND group; ^#^*p < 0.01*, compared to MNDQ group); (**C**) Body weights *vs*. time; (*p < 0.01, compared to DOX group) (**D**) Images of H&E-stained sections of tumor excised from subcutaneous tumor-bearing mice at the end of the experiment: saline group(a), MNQ group (b), DOX group(c), (DOX + QUT) group (d), MND group (e), MNDQ group (f), BNDQ group (g).

After studying the anticancer activity of BNDQ *in vitro*, we proceeded to evaluate its anticancer efficacy *in vivo*. MCF-7/ADR cells were used to establish our tumor model. When the tumor volume grew to about 50 mm^3^, mice were divided into seven groups and administrated intravenously once every three days for a total of seven doses with saline, DOX, DOX + CUR, MND, MNQ, MNDQ, or BNDQ, respectively. As shown in Figure 9A, at the end of the test, the tumor volumes of saline, DOX, DOX + CUR, MND, MNQ, MNDQ, and BNDQ were were 850 ± 68 mm^3^, 663 ± 35 mm^3^, 645 ± 56 mm^3^, 800 ± 62 mm^3^, 520 ± 68 mm^3^, 386 ± 42 mm^3^ and 212 ± 42 mm^3^, respectively. The average tumor volume of the BNDQ-treated group was the smallest among all the treated groups, demonstrating the superior inhibition efficacy of biotin-decorated nanoparticles *in vivo*. This result was further demonstrated by the BNDQ-treated group showing the lowest tumor weight of (Figure 9B). Moreover, the antitumor efficacy of BNDQ was in good agreement with the tendencies of both the *in vitro* cytotoxicity and cellular uptake models. The changes in body weight were measured simultaneously (Figure 9C) for preliminarily evaluation of the *in vivo* safety of formulations. The treatment with DOX and (DOX+QUT) showed a clear loss of body weight during the course of the study, which means that these treatment groups exhibited toxicity in mice at the given dosage. In contrast, none of the nanoparticle-treated groups caused weight loss, which implies that they could reduce the adverse effects of DOX. To further evaluate the *in vivo* antitumor activities of all the treatment groups, the tumors excised from the mice were sectioned for H&E staining. As presented in Figure 9D, various degrees of tissue necrosis were observed, chromatins were concentrated and distributed around the edge, and nuclei became pyknotic or absent, in the DOX-loaded nanoparticle-treated groups (MND, MNDQ, BNDQ). The necrosis area in the BNDQ-treated group was the largest among all the tested groups, which further demonstrated that the BNDQ resulted in the best antitumor efficacy *in vivo*.

Drug-resistant cancer cells that overexpress ATP-binding cassette transporters such as P-gp can facilitate the efflux of chemotherapeutic drugs [40–43]. To solve this problem, many P-gp inhibitors such as verapamil, cyclosporine, curcumin and QUT were used together with chemotherapeutic drugs [44–45]. *In vitro* these combinations show good inhibition effects on drug resistant cancer cells, but *in vivo* the anticancer efficacies of these combinations were not as good as expected. Because the P-gp inhibitor and chemotherapeutic drug have different biopharmaceutical and pharmacokinetic properties, they could not reach and exert an effect on the same cancer cell at the same time after systemic administration *in vivo*. Herein, to ensure P-gp inhibitor (QUT) and chemotherapeutic drug (DOX) could be simultaneously delivered in a targeted manner to tumor cells, we prepared biotin decorated poly(ethylene glycol)-b-poly(ε-caprolactone) nanoparticles encapsulating QUT and DOX (BNDQ). The superior advantages of BNDQ in minimizing cancer drug resistance *in vivo* could be attributed to the combination of: (1) it allowed higher tumor accumulation of drug by the EPR effect and biotin receptor mediated tumor-targeting ability; (2) improved cellular uptake of DOX *via* biotin receptor mediated endocytosis of BNDQ; (3) increased the intracellular concentration and retention of DOX in cancer cells showing MDR, by inhibiting both P-gp activity and expression, and thereby reducing the drug efflux rate.

## MATERIALS AND METHODS

### Materials

QUT was purchased from Aladdin (Shanghai, China). Biotin-PEG_2k_-PCL_5k_ and MPEG_2k_-PCL_5k_ were obtained from Jinan Daigang Biotechnology Co., Ltd. (Jinan, China). Doxorubicin hydrochloride was purchased from Beijing Huafeng United Technology Co., Lid. (Beijing, China). 3-(4,5-dimethylthiazol-2-yl)-2,5-diphenyltetrazolium bromide (MTT) and dimethyl sulfoxide (DMSO) were obtained from Solarbio Science & Technology (Beijing, China). RPMI 1640 medium was purchased from Invitrogen Corporation (Grand Island, USA). Fetal bovine serum (FBS), Penicillin-streptomycin, 0.25% (w/v) trypsin, 0.03% (w/v) Ethylene Diamine Tetraacetic Acid (EDTA) solution and phosphate-buffered saline (PBS) was purchased from Gibco BRL (Gaithersburg, MD, USA). Female BALB/c nude mice aged 6 weeks (18~22 g) were purchased from SLRC Laboratory Animal Company (Shanghai, China). All animals received care in compliance with the guidelines outlined in the Guide for the Care and Use of Laboratory Animals and all procedures were approved by the Animal Care and Use Committee of Sun Yat-Sen Memorial Hospital, Sun Yat-Sen University.

### Preparation of nanoparticles

Doxorubicin hydrochloride was first deprotonated using the previous reported method to make it hydrophobic before the preparation of the nanoparticles encapsulating DOX [46]. Then, BNDQ and MNDQ were prepared by a thin film hydration method [25, 26]. Briefly, 5 mg of DOX and 10 mg of QUT were mixed with 100 mg of carrier in 30 mL solutions containing methanol and chloroform (3/7, v/v), followed by sonication at room temperature for 30 min. Next, the organic solvents were removed by rotary evaporation to obtain a drug/polymer matrix. The matrix was further dried under high vacuum overnight to remove any remaining organic solvent. The matrix was hydrated with 20 mL of water using a probe-type sonicator (Xin Zhi Biotechnology Co. Ltd., China) at 200 w for 5 min. The resulting suspension was centrifuged at 4000 rpm for 20 min to remove any aggregated particles and unencapsulated drug. After centrifugation, nanoparticles were obtained and kept at 4°C for further use or lyophilization. The DOX-loaded nanoparticles (BND and MND), QUT-loaded nanoparticles (BNQ and MNQ) and empty nanoparticles were prepared in a similar manner without adding QUT, DOX, or drug, respectively.

### Particle size analysis and zeta potential measurement

The average particle sizes of the prepared nanoparticles were measured by dynamic light scattering (DLS) using a Zeta Size Nano-S(Malvern Instrument) at a detection angle of 173°C and the zeta potentials of the prepared nanoparticles were determined by the light scattering method using 90 Plus Particle Size Analyzer(BC Haven Instruments Corporation). All measurements were repeated three times at 25°C.

### Morphological analysis

The morphology of BNDQ was observed by transmission electron microscopy (TEM, JEM-2100/INCA OXFORD). A drop of diluted solution of BNDQ was placed on a copper grid and dried before measurement.

### Measurement of encapsulation efficiency and drug loading content

The encapsulation efficiencies and drug loading contents of the drug-loaded nanoparticles were determined by the high performance liquid chromatography (HPLC) method, using a Shimadzu HPLC system equipped with a SPD-10 Avp detector, a LC-10 ADvp pump and a Diamonsil C_18_ reversed phase column (4.6 mm × 250 mm, 5 μm). Briefly, 10 mL acetonitrile was added to 1 mL of drug-loaded nanoparticle solution to disrupt the structure of the nanoparticle. The mixed solution was dried under nitrogen and redissolved in 5 mL acetonitrile. To measure the content of DOX in the acetonitrile solution, the mobile phase was prepared by mixing acetonitrile and water (72/28, v/v), adjusted to pH 3.0 with phosphoric acid, and pumped at a flow rate of 1.0 mL/min. The eluent was monitored at a wavelength of 254 nm. For detection of QUT, the mobile phase was composed of acetonitrile, 10 mM ammonium acetate buffer and methanol (32/48/20, v/v/v). The mobile phase was pumped at a flow rate of 1.0 mL/min. The detection wavelength for QUT was 370 nm. The encapsulation efficiency (EE%) and drug loading content (DL%) were calculated using equations (1) and (2) as follows:

$$\text{EE}\%=\frac{\text{weight of DOX or QUT in nanparticles}}{\text{weight of feeding DOX or QUT}}\times 100\%$$ (1)

$$\text{DL}\%=\frac{\text{weight of DOX or QUT in nanparticles}}{\text{weigth of the drug }-\text{ loaded nanoparticles}}\times 100\%$$ (2)

### *In vitro* drug release study

The *in vitro* release behaviors of DOX and QUT from the drug-loaded nanoparticles were studied by a dialysis method. Briefly, 10 mg freeze-dried powders of drug-loaded nanoparticles were dissolved in 5 mL release medium consisting of PBS (pH 7.4) with 0.5% Tween 80 and then placed in a dialysis bag with the molecular weight cut-off of 3500 Da (Snakeskin, Pierce, USA). The dialysis bag was suspended in a tube containing 25 mL of the release medium. The tube was placed in a shaking water bath at 37°C with a shaking speed of 120 rpm. At selected time intervals, the release medium in the tube was completely drawn and replaced with fresh release medium. The amounts of DOX and QUT in the release medium were determined by the methods in the “Measurement of Encapsulation Efficiency and Drug Loading Content” section.

### *In vitro* cytotoxicity assay

Briefly, MCF-7 or MCF-7/ADR cells in their logarithmic growth were seeded in 96-well plates at a density of 5 × 10^3^ cells/well. Following overnight incubation, cells were treated with free DOX, (DOX + QUT), MND, MNDQ and BNDQ at various drug concentrations for 48 h. Upon completion of the incubation period, the medium containing drug was aspirated. The cells were washed with PBS (pH 7.4), subsequently incubated with 200 μL medium containing 0.5 mg/mL MTT for another 4 hours. The medium in the well was carefully removed and replaced with 0.2 mL of DMSO. The absorbance values of the DMSO solutions were measured at 570 nm by an ELISA plate reader (Varioskan Flash).

Cells treated with drug-free medium were used as control. In order to evaluate the cytotoxicities of the drug carriers, the cytotoxicities of the drug-free nanoparticles against MCF-7 and MCF-7/ADR cells were also conducted using the same method as mentioned above. To investigate the effects of free biotin on the *in vitro* cytotoxicities of BNDQ and MNDQ, MCF-7 or MCF-7/ADR cells were pre-incubated with 2 mM free biotin for 4 h before the cells were exposed to series of concentrations of BNDQ or MNDQ. Then the cytotoxicities of these nanoparticles were assessed using the above method.

### Cellular uptake studies

MCF-7 cells or MCF-7/ADR cells were seeded at a density of 5 × 10^4^ cells/well in 24-well plates and allowed to attach overnight. The cells were then exposed to fresh media containing different DOX formulations (the concentration of DOX was 5 μg/mL) for 4 h at 37°C. Subsequently, the media were removed and the cells were washed with PBS (pH 7.4) three times before observation under the fluorescence microscope (DMI 6000B, LeiCa). The extent of cellular uptake in different DOX formulations was further quantitatively determined by flow cytometry. Briefly, MCF-7 cells or MCF-7/ADR cells were seeded in 12-well plates (1 × 10^6^ cell/well) and allowed to attach overnight. Then the cells were incubated at 37°C with fresh media containing different DOX formulations at a DOX concentration of 5 μg/mL for 0.5, 2, 4 or 8 h. At the end of the treatment period, the cells were washed three times with ice-cold PBS and digested with 0.25% trypsin. Then, the cells were resuspended in 0.5 mL pre-cold PBS, measured by flow cytometer (FACS Calibur, BD, USA) and analyzed with Cell Quest Software through fluorescence channel 2 (FL_2_). To study whether the cellular uptake of BNDQ is mediated *via* the biotin receptor, a competitive experiment in presence of free biotin was performed. MCF-7 cells or MCF-7/ADR cells were treated with 2 mM free biotin for 1.5 h before incubation with BNDQ and MNDQ and then subjected to above protocol.

### DOX efflux study

To investigate the efflux of DOX in both MCF-7 and MCF-7/ADR cells after incubation with different DOX formulations, the cells were seeded in 12-well plates (5 × 10^4^ cells/well) and allowed to attach overnight. Next, they were incubated with fresh media containing different DOX formulations (the concentration of DOX was 10 μg/mL) for 4 h at 37°C. After washing three times with PBS, cells were further incubated with drug-free media at 37°C for another 0.5, 1, 2 or 4 h. At the end of the incubation, the cells were washed three times with ice-cold PBS, digested with 0.25% trypsin, and suspended in 0.5 mL pre-cold PBS. The amounts of DOX retained in cells were determined by flow cytometry (FACS Calibur, BD, USA) and analyzed with Cell Quest Software through FL_2_.

### Analysis of the P-gp activity

A method reported by Zhang *et al.* [47] was used in this study to analysis the P-gp functional activity of cancer cells. Briefly, MCF-7/ADR or MCF-7 cells were seeded into 12-well plates at a density of 1 × 10^5^ cell/well for MCF-7/ADR cells, and 0.5 × 10^5^ cell/well for MCF-7 cells. Following overnight incubation, the cells were treated with DOX, (DOX+QUT), MND, MNDQ or BNDQ at a DOX concentration of 5 μg/mL for 4 h. Hoechst 33342 (4 μM) was added to each well and incubated for another 0.5 h. Then the cells were washed three times with cold PBS and lysed with 1 mL of 0.1% Triton-X 100. A fluorescence microplate reader (BioTek, Winooski, VT) was used to determine the content of Hoechst in the cell lysates. The excitation and emission wavelengths were set at 370 and 450 nm, respectively. The protein concentrations of the cell lysates were quantified by BCA assay (Pierce, Rockford, IL) following the manufacturer's protocol.

### Evaluation of P-gp expression

MCF-7/ADR or MCF-7 cells were seeded into 6-well plates (2 × 10^5^ cell/well, for MCF-7/ADR cells; 1 × 10^5^ cell/well, for MCF-7 cells) and allowed to attach overnight. Next the media was replaced with fresh media containing DOX, (DOX+QUT), MND, MNDQ or BNDQ (the concentrations of DOX was 5 μg/mL). The cells were cultured for another 48 h at 37°C. Then the cells were trypsinized, collected, and resuspended in 0.1 mL PBS (pH 7.4). PE-conjugated mouse anti-human monoclonal antibody against P-gp was used to label the above cells according to the manufacture's protocol, and nonspecific labeling was corrected by use of an isotype control. The fluorescence intensities of the cells were determined by a FACS Caliber (Beckton Dickinson, USA).

### Determination DOX accumulation in tumors

To study whether coencapsulation of DOX and QUT into the same nanoparticle could enhance the DOX accumulation in tumor tissue, a method reported by Park *et al.* [48] was used in our study. Briefly, MCF-7/ADR cells (6 × 10^6^ cells in 0.2 mL 1640 culture medium) were injected subcutaneously into the right axilla of female BALB/c nude mice. After the tumor volumes reached about 500 mm^2^, the mice were divided into five groups (each group have 4 mice) by randomly, and then administered through the tail vein either free DOX, (DOX+QUT), MND, MNDQ or BNDQ, at the DOX dosage of 5 mg/kg. The tumors were removed after 12 h of administration. The collected tumors were homogenized for 1 min using a hand-held tissue homogenizer and suspended in cold PBS (pH 7.4). The suspensions were centrifuged at 15,000 rpm for 15 min to clarify the tissue extracts. The amount of extracted tumor tissue was quantified by BCA protein assay and fluorescence intensities of DOX were monitored using the microplate reader.

### *In vivo* antitumor activity

Female BALB/c nude mice (6 weeks old, body weight 18~22 g) were purchased from SLAC laboratory animal company (Shanghai, China) and maintained in a specific pathogen-free (SPF) animal lab. All animal care and procedures for animal experiments were approved by the Animal Care and Use Committee of Sun Yat-Sen Memorial Hospital, Sun Yat-Sen University. MCF-7/ADR cells in their exponential growth phase were harvested by centrifugation and resuspended in fetal bovine serum-free PRMI 1640 medium to form cell suspensions with a density of 3 × 10^7^/mL. 100 mL MCF-7/ADR cell suspension was injected subcutaneously into the right axilla of nude mice. When tumors had grown to about 50 mm^3^, this day was designated as day 0, and the mice were randomly divided into 7 groups (6 mice per group): Saline, DOX, (DOX + QUT), MNQ, MND, MNDQ and BNDQ (DOX dosage: 5 mg/kg). The injections were carried out on days 0, 2, 5, 8, 11, 14, 17 and 20 *via* the tail vein. In order to evaluate the treatment efficacy and safety, the tumor volume and body weight were measured one day after administration. The tumor volume was calculated using the formula V = ab^2^/2, where a and b are the major and minor axes of the tumors measured by caliper, respectively. Tumors were collected for H&E staining one day after the final treatment.

## CONCLUSIONS

In conclusion, the tumor-targeting dual drug-loaded nanoparticles BNDQ have significant advantages compared to treatment with the free drug combination (DOX + QUT), single drug loaded nanoparticles (BND, BNQ) or non-biotin decorated nanoparticles (MNDQ, MND, MNQ), in dealing with the drug resistant breast cancer cells, MCF-7/ADR cells. The significant advantages observed may be partially attributed to the clear increase in the intracellular concentration and retention of DOX, which occurred via facilitating both the cellular drug uptake and reducing the drug efflux rate in MCF-7/ADR cells, and by the inhibition of both P-gp activity and expression in MCF-7/ADR cells. Furthermore, BNDQ significantly enhanced the *in vivo* anticancer efficacy of DOX in a drug-resistant MCF-7/ADR xenograft model. These results indicate that biotin receptor-mediated tumor-targeting nanoparticles encapsulating chemotherapeutic drug and chemosensitizer could provide specific and efficient formulations to reverse drug resistance in human breast cancer.
